# A Pilot Study Exploring Paraoxonase-1 Tissue Protein Expression and Circulating Levels in Bladder Cancer

**DOI:** 10.3390/antiox15020198

**Published:** 2026-02-02

**Authors:** David Parada, Alina-Iuliana Onoiu, Simona Iftimie, Jordi Camps, Francesc Riu, Antoni Castro, Jorge Joven

**Affiliations:** 1 Department of Pathology, Hospital Universitari de Sant Joan, Institut d’Investigació Sanitària Pere Virgili, Universitat Rovira i Virgili, Av. Dr. Josep Laporte 2, 43204 Reus, Spain; 2 Unitat de Recerca Biomèdica, Hospital Universitari de Sant Joan, Institut d’Investigació Sanitària Pere Virgili, Universitat Rovira i Virgili, Av. Dr. Josep Laporte 2, 43204 Reus, Spain; 3 Autoimmunity, Infection and Thrombosis Research Group (GRAIIT), Department of Internal Medicine, Hospital Universitari de Sant Joan, Institut d’Investigació Sanitària Pere Virgili, Universitat Rovira i Virgili, Av. Dr. Josep Laporte 2, 43204 Reus, Spain

**Keywords:** bladder cancer, immunohistochemistry, methylthioadenosine phosphorylase, MTAP, paraoxonase-1, PON1

## Abstract

Paraoxonase-1 (PON1) is considered a liver-derived antioxidant enzyme circulating bound to high-density lipoproteins, with limited evidence of protein expression in human urothelial tissue. Its role in bladder cancer remains unexplored. Methylthioadenosine phosphorylase (MTAP), an enzyme related to tumor aggressiveness, may interact with oxidative and metabolic stress pathways relevant to tumor progression. We conducted an exploratory study integrating immunohistochemistry, serum biochemistry, and PON1 genotyping in 39 patients with low-grade (LGUC) or high-grade urothelial carcinoma (HGUC). Both PON1 and MTAP showed reduced expression in high-grade tumors, with MTAP reduction being more pronounced and consistent than that of PON1. Serum PON1 concentrations and activities were slightly reduced in HGUC compared with LGUC and controls. Genotype frequencies were similar between patients and controls, and polymorphisms influenced serum enzymatic activity similarly in both groups. Correlations between tissue and serum PON1 did not reach significance, although descriptively low tissue expression aligned with low serum levels. This study provides initial evidence of intratumoral PON1 expression in bladder cancer and suggests that combined PON1/MTAP immunohistochemical assessment may reflect tumor grade and biological behavior. Larger functional studies are needed to clarify their mechanistic and clinical relevance.

## 1. Introduction

Bladder cancer is among the most prevalent malignancies of the urinary tract worldwide. This disease represents a significant public health concern due to its high incidence, substantial recurrence rate, and potential for invasive progression [1]. Despite advances in early detection, surgical techniques, and adjuvant therapies, long-term outcomes remain suboptimal for a considerable proportion of patients [2]. The etiology and clinical course of bladder cancer are strongly influenced by chronic exposure to oxidative stress and persistent inflammation, which can arise from both endogenous metabolic processes and environmental insults. Exposure to carcinogenic compounds such as those derived from tobacco smoke and aromatic amines leads to the generation of reactive oxygen species and the subsequent oxidation of lipids and proteins within the urothelium [3,4]. These molecular alterations highlight the potential value of investigating endogenous antioxidant systems, such as paraoxonase-1 (PON1), as modulators of the oxidative environment in bladder cancer.

PON1 is a member of the paraoxonase family, which also includes PON2 and PON3 [5,6]. While PON2 is primarily regarded as a tissue-associated enzyme responsible for local antioxidant protection and has been linked to several types of cancer [7,8,9], PON1 is generally considered to act in the circulation, bound to high-density lipoproteins (HDL).

Several studies, however, have reported detectable PON1 protein expression in various tissues in rodents and humans [10,11,12], but the presence and distribution of PON1 in bladder tissue, particularly in the context of cancer, remain undescribed. Understanding whether PON1 is expressed in the tumor microenvironment is therefore crucial to elucidate its potential role as a modulator of oxidative stress at the tissue level, complementing its systemic activity in circulation.

PON1 exerts its protective effects through multiple enzymatic activities, including lactonase, paraoxonase, and arylesterase functions, which allow it to degrade lipid peroxides and detoxify organophosphate xenobiotics such as paraoxon and phenylacetate. The enzyme’s activity and serum concentration are strongly influenced by genetic polymorphisms, most notably the PON1 Q192R (rs662) and L55M (rs854560) variants. The 55M allele is associated with lower enzyme stability and activity, partly due to linkage disequilibrium with promoter variants, whereas the 192 polymorphism affects substrate-specific catalytic efficiency [5]. Altered serum PON1 concentration or activity has been implicated in several oxidation- and inflammation-related diseases, including cardiovascular disorders, liver disease, and multiple cancer types [5,6,13,14].

This pilot study was therefore designed to examine PON1 protein expression in bladder tumor tissue, clarify its cellular localization, and evaluate its systemic activity through serum measurements. In parallel, common PON1 polymorphisms (Q192R and L55M) were genotyped to explore their potential association with tissue and circulating enzyme levels. In addition to PON1, we assessed the expression of methylthioadenosine phosphorylase (MTAP), a metabolic enzyme frequently lost in urothelial and other cancers. MTAP loss has been associated with tumor progression and altered cellular metabolism, making it a relevant marker for histopathological evaluation [15]. Assessing MTAP alongside PON1 allows the characterization of tumor heterogeneity, including nuclear versus cytoplasmic staining patterns, providing complementary information on tissue-level alterations in both low- and high-grade tumors. By integrating tissue, circulation, and genetic data, this study provides preliminary insights into the role of PON1 in bladder cancer and its contribution to the oxidative balance within the tumor microenvironment.

## 2. Materials and Methods

### 2.1. Participants

This retrospective and descriptive cohort study was conducted at the Hospital Universitari de Sant Joan (Reus, Spain). A total of 39 patients in whom urinary bladder cancer had been confirmed by transurethral resection were included. Cases of urothelial carcinoma diagnosed between January 2002 and December 2013 were retrieved from the Department of Pathology archives. Patients were excluded if they had severe alcohol abuse, psychiatric disease, or evidence of liver impairment.

Both tissue-level and serum-level measurements of PON1 were performed to address complementary biological questions. Immunohistochemistry (IHC) provides information on the cellular localization and presence of PON1 within bladder tumor tissue, whereas serum PON1 concentration and enzymatic activities reflect systemic circulating levels. The potential relationship between tumor expression and circulating PON1 is currently unknown. In addition, common PON1 gene polymorphisms (Q192R and L55M) were analyzed to explore their possible influence on enzymatic activity and serum concentration, as well as to assess potential differences between healthy individuals and patients. These approaches were included in an exploratory manner to capture distinct aspects of PON1 biology in bladder cancer.

Clinical information was obtained from electronic medical records, including age, sex, and relevant laboratory data. All patients underwent routine clinical follow-up, with their records and number of cancer recurrences reviewed for at least five years after the initial diagnosis.

For the biochemical and genetic analyses, a control group consisting of 47 healthy individuals was included. The control participants were matched to the patient group for age and sex distribution. They exhibited no clinical or biochemical evidence of infectious diseases, diabetes mellitus, renal insufficiency, hepatic disorders, cancer, or neurological conditions. These samples were obtained from a study conducted by the Department of Epidemiology at our university, which focused on a healthy reference population. Participants were identified through telephone interviews based on municipal census data. Subsequently, they underwent a standardized clinical interview and basic laboratory testing to confirm the absence of any health conditions that could influence the study outcomes [16].

The study protocol was reviewed and approved by the Ethics Committee (Institutional Review Board) of the Hospital Universitari de Sant Joan, and all participants provided written informed consent in accordance with the principles of the 1964 Helsinki Declaration and its subsequent amendments.

### 2.2. Histopathological Characteristics

Tumor grade was categorized as low-grade urothelial carcinoma (LGUC) or high-grade urothelial carcinoma (HGUC) based on the degree of cellular and architectural atypia [17]. Low-grade tumors display relatively well-organized architecture and mild cytologic abnormalities, whereas high-grade tumors show marked atypia, disorganized growth, and a higher likelihood of invasion and progression. Tumor invasion was classified as follows: muscle-invasive urothelial carcinoma (MUSC), in which the tumor invaded the muscle layer of the bladder; T1 subepithelial connective tissue invasion (TCSE), where the tumor invaded the subepithelial connective tissue without reaching the muscle layer; and T1 subepithelial connective tissue invasion (non-flat) (TCSE NF), where the invasion of the subepithelial tissue was more irregular or nodular [18].

### 2.3. Histological and Immunohistochemical Study

Formalin-fixed, paraffin-embedded bladder biopsy specimens from each patient were sectioned at 2 µm thickness and stained with hematoxylin and eosin (H&E). An experienced pathologist evaluated the tumor histological pattern, grade, and pathological stage.

For immunohistochemical analysis, an additional 2 µm section was mounted on a silanized slide and processed on a VENTANA^®^ Benchmark ULTRA/LT automated immunostainer (Ventana Medical Systems, Oro Valley, AZ, USA). The standardized protocol was applied for PON1 and MTAP detection (Hoffmann-La Roche, Basel, Switzerland), including antigen retrieval with a pH 9 buffer and visualization using the OptiView^®^ DAB IHC Detection Kit (VENTANA^®^). The primary antibodies were incubated for 32 min at room temperature. Immunoreactivity was revealed with diaminobenzidine (DAB), counterstained with Meyer’s hematoxylin, and examined under an Olympus BX41 (Olympus Corp. Tokyo, Japan) light microscope at magnifications ranging from 2× to 60×.

Positive IHC staining for PON1 and MTAP was defined as the presence of cytoplasmic and/or nuclear reactivity in tumor cells. Appropriate positive and negative controls were included in each staining run to ensure specificity and technical reliability. All tumor areas were systematically evaluated. IHC assessment was performed using a semi-quantitative approach based on previously published criteria [19,20,21,22], incorporating both the proportion of positive cells and staining intensity. The percentage of positive tumor cells was categorized as 0% (negative), 1–10%, 11–50%, 51–80%, or >80%. Cytoplasmic staining intensity was graded as absent (0), weak (1+), moderate (2+), or strong (3+), using the reactivity of positive controls as an internal reference for intensity assessment. Microscopic evaluation was performed at magnifications ranging from 4× to 20×.

### 2.4. Biochemical Measurements

Blood samples were collected after an overnight fast into anticoagulant-free tubes for serum preparation or into K_2_-EDTA tubes for genetic analyses. Aliquots were stored at −80 °C until batched analyses. Serum paraoxonase activity (PARX) was assessed by monitoring the hydrolysis of paraoxon at 410 nm and 37 °C in 0.05 mmol/L glycine buffer (pH 10.5) containing 1 mmol/L CaCl_2_ [23]. Paraoxon substrate was freshly prepared for each batch. Due to its toxicity and volatility, all measurements were conducted in a fume hood with appropriate personal protective equipment. Serum lactonase activity (LACT) was determined by measuring the hydrolysis of 5-thiobutyl butyrolactone (TBBL) in a reaction mixture containing 1 mmol/L CaCl_2_, 0.25 mmol/L TBBL, and 0.5 mmol/L 5,5′-dithio-bis-2-nitrobenzoic acid in 0.05 mmol/L Tris–HCl buffer (pH 8.0) [24]. Changes in absorbance were monitored at 412 nm. PARX and LACT activities were expressed in U/L. Serum PON1 concentrations were measured by a previously established in-house ELISA using antibodies against the CRNHQSSYQTRLNALREVQ peptide of mature human PON1 [25]. Serum samples were diluted in a 0.05 M sodium carbonate buffer and incubated overnight at room temperature. Plates were blocked with PBS containing 1% BSA, incubated with a primary rabbit anti-PON1 antibody, and then incubated with a horseradish peroxidase-conjugated secondary antibody. Color was developed using 3,3′,5,5′-tetramethylbenzidine, and the reaction was stopped with 2 M H_2_SO_4_.

### 2.5. DNA Extraction and Genotyping

For genotyping analyses, whole-blood samples were centrifuged at 1500× *g* and 4 °C. The buffy coat was isolated and incubated overnight with a cell lysis buffer. Proteins were then removed by adding a protein precipitation solution and centrifuging the mixture; the pellet was discarded, and the supernatant was retained. DNA was precipitated by adding cold isopropanol, washed, and finally resuspended in nuclease-free water.

DNA concentration and purity were determined using a NanoDrop™ spectrophotometer (Thermo Fisher Scientific, Waltham, MA, USA), with quality assessed by the 260/280 nm absorbance ratio. The PON1 Q192R and L55M polymorphisms were genotyped using Applied Biosystems TaqMan™ SNP Genotyping Assays. Approximately 2 μg of DNA was dispensed into each well of a 384-well plate and allowed to dry overnight. The assay master mix and other kit components were then added to the wells. The probes, designed to bind specifically within the minor groove of the DNA helix, stabilized the probe–template complex and enabled accurate discrimination between alleles.

### 2.6. Statistical Analyses

Quantitative data are presented as medians and 95% confidence intervals, and differences were assessed with the Mann–Whitney U test. Qualitative data are shown as numbers and percentages, and differences were evaluated with the χ^2^ test. Correlations were calculated by using the Spearman rho test. We employed the IBM SPSS Statistics for Windows package, Version 25.0 (IBM Corp., Armonk, NY, USA).

## 3. Results

### 3.1. Histopathological Results

The study included 34 (87%) men and 5 (13%) women, with a mean age of 68 years (62–75 years). Most high-grade tumors displayed an infiltrative growth pattern, whereas low-grade tumors were predominantly confined to the mucosa. High-grade tumors more often involved the subepithelial connective tissue, including non-focal cases, and the muscularis propria, while low-grade lesions largely remained limited to the mucosa.

The distribution of architectural patterns further distinguished the two groups. All low-grade tumors exhibited a papillary pattern, whereas high-grade tumors more frequently showed solid or mixed papillary–solid components, features rarely encountered in the low-grade group. Divergent histologic differentiation was also more prevalent in high-grade tumors, with adenocarcinomatous or squamous components identified in a notable proportion of cases. In contrast, such differentiation was absent in low-grade lesions. Similarly, concomitant carcinoma in situ was detected almost exclusively in high-grade tumors (Table 1).

Regarding clinical course, recurrence patterns at 1 year were similar across groups. Approximately half of the patients experienced a single recurrence, and multiple recurrences were less common and occurred at similar rates. At 5 years, the distribution of patients with no recurrence, a single event, or multiple recurrences remained analogous, with only minor variations and no meaningful differences between the two groups.

### 3.2. Immunohistochemical Findings

**Figure 1:**
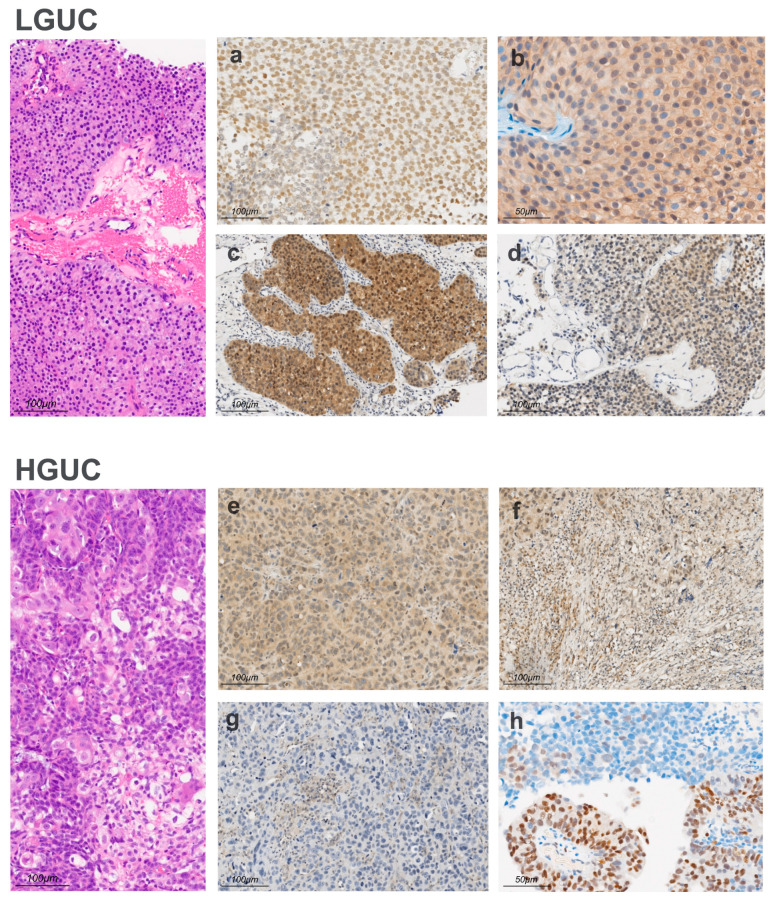
Representative sections of low-grade urothelial carcinoma (LGUC) and high-grade urothelial carcinoma (HGUC) are shown using hematoxylin–eosin (H&E) and immunohistochemical staining for paraoxonase-1 (PON1) and methylthioadenosine phosphorylase (MTAP). LGUC exhibits papillary fronds lined by orderly urothelial cells with mild nuclear enlargement and minimal cytologic atypia (H&E, 20×). PON1 staining in LGUC (**a**,**b**) shows diffuse cytoplasmic labeling with a largely homogeneous distribution across papillary structures. MTAP staining is variable, appearing strong and focal (**c**) or weak and diffuse (**d**). HGUC shows loss of polarity, marked nuclear pleomorphism, and numerous mitoses (H&E, 20×). In HGUC, PON1 immunostaining (**e**,**f**) is heterogeneous, with moderate cytoplasmic expression in some tumor regions and reduced or absent labeling in infiltrative foci. MTAP staining (**g**,**h**) shows extensive loss of expression, with only occasional residual positivity in isolated cells.

Immunohistochemical evaluation revealed distinct expression patterns for PON1 and MTAP between LGUC and HGUC (Figure 1). PON1 staining in LGUC was variable, with intensity ranging from weak (1+) to strong (3+) and displaying a combination of cytoplasmic and nuclear localization (Figure 1a,b). A complete absence of PON1 (score 0) was observed in 2 LGUC cases (7.1%). In HGUC, PON1 immunoreactivity was, overall, less intense than in LGUC (Figure 1e,f), although expression was still detectable in many tumors.

MTAP expression showed a contrasting pattern. LGUC cases showed variable MTAP staining, appearing strong and focal (c) or weak and diffuse (d), whereas HGUC frequently exhibited reduced or absent expression (Figure 1g,h). Complete loss of MTAP was more common in high-grade tumors than in low-grade ones.

### 3.3. Biochemical and Genetic Analyses

**Figure 2:**
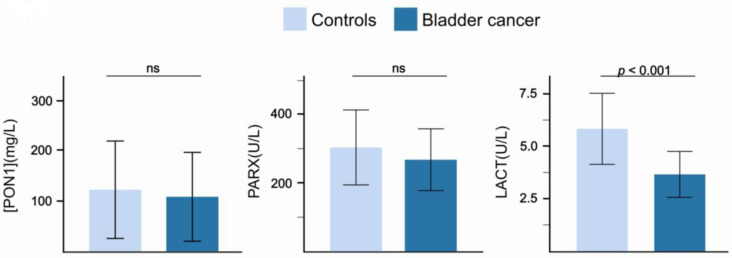
Paraoxonase-1-related variables in patients with bladder cancer and the control group. Abbreviations: LACT, lactonase activity; ns, not significant; PARX, paraoxonase activity; [PON1], paraoxonase-1 concentration.

Patients had serum PON1 concentrations and PARX activities comparable to those of the control group, whereas LACT activities were lower (Figure 2). When stratified by tumor grade, median serum PON1 concentrations and paraoxonase activities were numerically lower in high-grade patients compared with low-grade patients and controls, but these differences did not reach statistical significance (Table 2).

**Figure 3:**
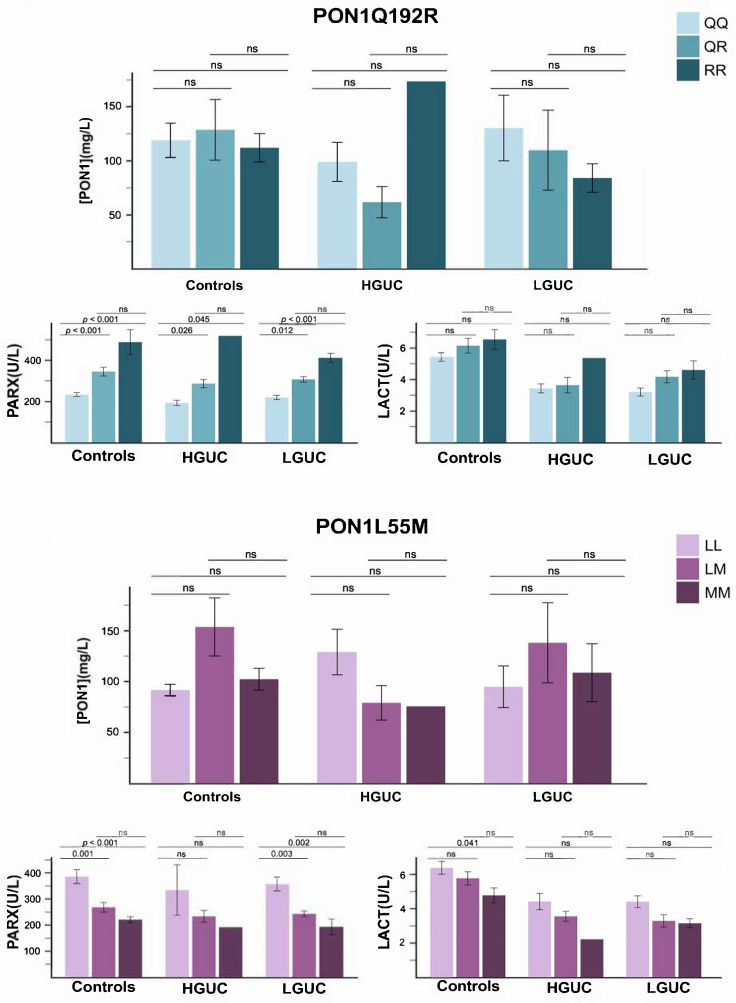
Paraoxonase-1-related variables in patients with bladder cancer and the control group, segregated by selected paraoxonase-1 gene polymorphisms and the grade of cancer. Abbreviations: HGUC, high-grade urothelial carcinoma; LACT, lactonase activity; LGUC, low-grade urothelial carcinoma; ns, not significant; PARX, paraoxonase activity; [PON1], paraoxonase-1 concentration.

No significant differences were observed between patients and controls in the frequencies of the PON1 Q192R and L55M genotypes (Table 2). Moreover, the effect of these polymorphisms on PON1-related variables was similar in both groups. Specifically, the Q192R and L55M variants significantly influenced PARX activity in patients and controls alike, whereas their impact on LACT and PON1 concentration was minimal or negligible (Figure 3).

### 3.4. Relationships Between Cellular PON1 Expression and Circulating PON1-Related Variables

**Figure 4:**
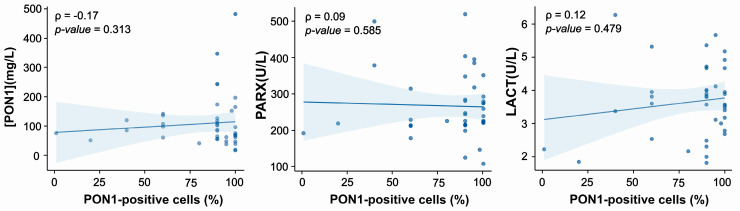
Relationship between the percentage of paraoxonase-1 (PON1)-positive cells in urothelial tissue and serum PON1-related variables. Abbreviations: LACT, lactonase activity; PARX, paraoxonase activity; [PON1], paraoxonase concentration. The solid line represents the regression line, and the shaded area indicates the 95% confidence interval.

Correlation analyses between the percentage of PON1-positive tumor cells and serum PON1 concentration or enzymatic activities did not reach statistical significance (Figure 4). Descriptively, cases with low tissue expression tended to show low serum levels, whereas cases with high tissue expression displayed a broader range of serum values. These observations are presented as exploratory, and no causal or mechanistic conclusions can be drawn.

## 4. Discussion

To our knowledge, this study provides the first evidence of detectable PON1 protein expression within human urothelial carcinoma cells. PON1 has traditionally been described as a liver-derived enzyme circulating bound to HDL [5], with limited protein expression reported in a few non-hepatic tissues. More recent studies in rodent and human tissues suggested a broader biological distribution [10,11,12,26,27]. However, information on PON1 protein expression in human urothelium remains minimal and inconsistently reported, which highlights the need for direct immunohistochemical evaluation. The Human Protein Atlas reports only weak or inconsistent PON1 staining in normal urothelial tissue [12]. Our immunohistochemical findings therefore extend the current understanding of PON1 biology, demonstrating that the enzyme is present in bladder tumor cells and exhibits patterns that vary with tumor grade. These findings underscore a potentially novel aspect of PON1 localization in the tumor microenvironment.

Our results raise the question of how PON1 appears within bladder tumor cells. Early mRNA expression studies failed to detect PON1 transcripts in most human non-hepatic tissues, suggesting that local synthesis outside the liver and intestine is minimal or below the sensitivity of these techniques [26,28]. In contrast, immunohistochemical and biochemical data indicate that PON1 protein can be detected in a variety of tissues in mammals. This discrepancy between mRNA and protein distribution raises the plausible scenario that circulating HDL-bound PON1 may be taken up or transferred to peripheral tissues.

Indeed, experimental evidence supports the concept that HDL-associated PON1 can redistribute to cell membranes and remain enzymatically active, implying a mechanism by which PON1 can be delivered from the circulation to peripheral cells. In this model, interaction with cell surface receptors facilitates the exchange of PON1 between HDL and the plasma membrane [29]. Consequently, the PON1 immunoreactivity observed in bladder tumor cells could reflect either local uptake of HDL-bound PON1 or, less likely, low-level synthesis that has not yet been detected by sensitive transcriptomic methods. Therefore, both local synthesis and uptake from the extracellular milieu remain plausible mechanisms, highlighting a potentially important aspect of PON1 biology in urothelial carcinoma.

PON1 expression in low-grade tumors tended to be stronger and more homogeneous. In contrast, high-grade tumors displayed weaker and more heterogeneous staining. Although speculative at this stage, this pattern may reflect altered antioxidant capacity or dysregulated cellular responses to oxidative stress during tumor progression, as reported in several types of cancer [30,31]. A similar trend was observed in the biochemical analysis, with high-grade patients showing slightly lower serum PON1-related variables than low-grade patients and controls. However, these differences were not statistically significant, and any apparent concordance with tissue-level changes should be interpreted with caution.

Beyond its presence, the potential functional role of PON1 within urothelial carcinoma cells warrants consideration. In the tumor context, intracellular PON1 could contribute to modulating local redox homeostasis, counteracting reactive oxygen species generated during metabolic stress, chronic inflammation, or exposure to environmental toxins [5,13]. This activity might influence tumor cell survival, proliferation, and resistance to oxidative insults. Additionally, PON1 could indirectly affect the tumor microenvironment by limiting oxidative modifications of extracellular lipids and proteins, thereby modulating signaling pathways involved in tumor progression [32]. These preliminary findings support the need for further studies to explore the interplay between PON1, oxidative stress, and tumor aggressiveness, which could uncover new therapeutic targets or biomarkers. [33].

Complementing these observations, analysis of common PON1 polymorphisms (Q192R and L55M) revealed similar genotype frequencies in patients and healthy volunteers. It should be emphasized that our study was not intended as a genetic association analysis, and the small sample size precludes formal conclusions regarding links between these variants and bladder cancer susceptibility or enzyme activity. Nevertheless, we determined these polymorphisms to ensure that cases and controls were matched for genotype, as differences in allelic frequencies could confound the interpretation of enzymatic activity in small cohorts, as previously discussed by our group [34]. In our dataset, the genotype distributions were comparable between patients and controls, suggesting that the modest tissue- or serum-level differences observed are likely attributable to disease-specific effects rather than inherited variation. This finding aligns with prior reports in other cancer types, where associations between these polymorphisms and disease risk or enzyme activity have been inconsistent and dependent on the investigated population [35,36,37]. Taken together, these results underscore that, while PON1 genotypes do influence enzymatic activity, the tissue- and serum-level alterations seen in bladder cancer may reflect tumor-specific regulation and local micro-environmental factors rather than genetic variation.

The observation that LACT activity, but not PARX or serum PON1 concentration, was significantly reduced in patients is biologically plausible and deserves specific consideration. PON1 is a promiscuous enzyme with the capacity to hydrolyze multiple substrates. However, converging experimental and structural evidence indicates that its ancestral and physiologically primary activity is that of a lactonase [24]. In contrast, hydrolysis of organophosphates such as paraoxon reflects a secondary catalytic function. Its efficiency is highly substrate-dependent and strongly modulated by common functional polymorphisms, particularly Q192R [5]. These variants markedly affect catalytic turnover toward paraoxon and introduce substantial interindividual variability, which may obscure disease-related differences. Lactonase activity, being less influenced by these polymorphisms, is considered a more robust indicator of the intrinsic functional status of PON1 in vivo. Regarding serum PON1 concentration, its regulation is complex and influenced by hepatic synthesis, HDL metabolism, inflammatory status, and genetic background [38]. Thus, subtle disease-related changes may be difficult to detect in small cohorts. Together, these considerations suggest that the selective reduction in lactonase activity may represent the most sensitive indicator of altered PON1 function in this setting, whereas paraoxonase activity and protein concentration may be confounded by genetic and metabolic variability.

An additional aspect of our study is the evaluation of MTAP expression, a well-characterized tumor suppressor gene involved in the methionine salvage pathway [39,40]. We observed strong and diffuse MTAP expression in low-grade tumors, but it was frequently reduced or absent in high-grade lesions. MTAP loss is a recognized molecular hallmark of aggressive behavior and is often co-deleted with CDKN2A at 9p21 [41,42,43]. Although our study was not designed to determine the genomic basis of MTAP loss, the differential expression between tumor grades supports its role as a marker of high-grade transformation in urothelial carcinoma.

PON1 and MTAP immunohistochemistry differ markedly between LGUC and HGUC, offering potential diagnostic and prognostic utility. Strong MTAP/PON1 staining favors low-grade classification, whereas weak/absent MTAP and heterogeneous PON1 suggest high-grade disease and may guide targeted surveillance. Indeed, MTAP loss has been reported to correlate with aggressive morphology and identifies candidates for clinical trials targeting methionine salvage [44]. Immunohistochemical assessment is compatible with routine diagnostics and requires minimal additional resources.

The immunohistochemical evaluation of PON1 and MTAP in this study provides insight into their potential utility as complementary markers in diagnostic, prognostic, and predictive frameworks for urothelial carcinoma. Bladder cancer is characterized by heterogeneous genomic and epigenomic alterations that influence tumor behavior and therapeutic response [45]. Recent evidence highlights the role of DNA methylation and hydroxymethylation profiles in disease progression, immune modulation, and biomarker development [46,47,48,49]. Within this context, PON1 and MTAP could enrich multiparametric panels by reflecting metabolic and epigenetic dysregulation, complementing established markers such as fibroblast growth factor receptor 3, the gene of tumor protein p53, and programmed death-ligand 1 [50]. Moreover, methylation-based assays, including Epicheck, are emerging as promising tools for early detection and risk stratification [47]. A key question for future research is whether PON1 expression is influenced by the tumor microenvironment, which could impact both its biological function and clinical relevance. Collectively, these considerations emphasize the need for integrative studies combining immunohistochemistry with genomic and epigenomic profiling to enhance diagnostic precision and prognostic assessment in urothelial carcinoma.

Finally, we examined whether cellular PON1 expression was associated with circulating PON1-related measures. Although our dataset was too small to support robust statistical modeling, the descriptive analysis suggested a potential non-random pattern linking tissue and serum PON1. Specifically, cases with low percentages of PON1-positive cells in the tumor consistently exhibited low serum PON1 concentrations, whereas cases with high tissue positivity showed a broader range of serum values. These observations are preliminary and descriptive, and they raise the possibility that a minimum level of intracellular PON1 expression could contribute to sustaining circulating PON1 levels, while additional factors (genetic, metabolic, or tumor-related) may influence the upper range of serum concentrations. Interpretation should therefore remain cautious, and confirmation in larger cohorts is needed.

## 5. Limitations and Future Perspectives

This study has several limitations inherent to its retrospective and exploratory design. The sample size was relatively small, limiting statistical power and precluding robust multivariate analyses. Consequently, some descriptive trends, such as the relationship between tissue-level and circulating PON1, should be interpreted cautiously.

Although patients and controls were matched for age and sex, other potential confounding factors known to influence PON1-related variables, such as smoking status, alcohol consumption, and metabolic comorbidities, could not be systematically evaluated or stratified due to the limited cohort size.

Another limitation is the absence of adjacent non-tumoral bladder tissue for PON1 assessment. All tissue samples were obtained during routine diagnostic biopsies, which are targeted to visible lesions rather than to histologically normal mucosa. As a result, a systematic comparison between tumoral and non-tumoral urothelium was not possible.

Moreover, the study was not designed to establish causal or mechanistic links between PON1 expression, MTAP status, and tumor progression. MTAP was included as a well-established reference marker of tumor aggressiveness to contextualize PON1 findings within a known pathological framework. Any potential functional interaction between PON1 and MTAP remains speculative, and conclusions regarding their relationship should be interpreted strictly in a descriptive and hypothesis-generating context.

Semi-quantitative immunohistochemical scoring was performed by a single pathologist, albeit with extensive experience in uropathology. Routine implementation of these assessments in clinical practice would ideally require automated digital image analysis. However, the marked heterogeneity of PON1 and MTAP expression across tumor regions currently limits the reliability of such automated approaches. Future studies should employ optimized image analysis tools to enhance the reproducibility and objectivity of the measurements.

Additionally, the study did not include p16 immunostaining, which could have served as a complementary marker to validate MTAP loss and its association with 9p21 deletion. Tissue availability in this retrospective cohort precluded further analyses. Future prospective studies should incorporate p16 and other relevant markers to strengthen the biological interpretation of MTAP alterations.

Future research should address these limitations by incorporating larger, multicenter cohorts and quantitative approaches for both tissue and serum PON1 measurements. Investigating the interplay between clinical variables, such as smoking history and comorbidities, and PON1 levels could provide additional context and help identify patient subgroups with distinct biological or prognostic profiles.

Furthermore, developing a composite risk score integrating clinical history, laboratory data, and pathological features could help identify patients at higher risk of disease progression. Such a score could guide surveillance intensity, distinguishing those who may benefit from more frequent cystoscopic follow-up from those suitable for standard annual monitoring. Within this framework, combined PON1 and MTAP immunohistochemical assessment in tumor biopsies may further refine stratification, offering a translational tool for personalized management and follow-up strategies in bladder cancer.

## 6. Conclusions

This study should be viewed as a pilot, hypothesis-generating investigation rather than as a definitive validation of PON1 or MTAP as clinical biomarkers. By providing the first systematic immunohistochemical characterization of PON1 in human urothelial carcinoma and contextualizing it alongside MTAP, a well-established marker of tumor aggressiveness, our results define preliminary expression patterns associated with tumor grade and highlight biologically plausible links with metabolic and epigenetic dysregulation. Although the limited cohort size and retrospective design preclude mechanistic or outcome-based inferences, the data establishes a rationale for future prospective studies integrating paired normal and tumoral tissues, molecular profiling, and functional approaches to determine the clinical and biological significance of PON1 in bladder cancer. We believe that, within this clearly stated exploratory framework, the present work provides useful proof of concept and a foundation for subsequent translational research. 

## Figures and Tables

**Table 1:** Histopathological characteristics.

Characteristic	Type	HGUC (*n* = 13)	LGUC (*n* = 26)	*p*-Value
Pattern	Papillary	7 (53.8)	26 (100.0)	0.001
	Solid	3 (23.1)	0 (0.0)	
	Papillary and solid	3 (23.1)	0 (0.0)	
Invasion	No	3 (23.1)	26 (100.0)	<0.001
	Yes	10 (76.9)	0 (0.0)	
Invasion degree	No invasion	3 (23.1)	26 (100.0)	<0.001
	MUSC	4 (30.8)	0 (0.0)	
	TCSE	4 (30.8)	0 (0.0)	
	TCSE NF	2 (15.3)	0 (0.0)	
Divergent differentiation	No	10 (76.9)	26 (100.0)	0.031
	Yes	3 (23.1)	0 (0.0)	
In situ carcinoma	No	10 (76.9)	26 (100.0)	0.031
	Yes	3 (23.1)	0 (0.0)	
PPC (%)		90 (70–100)	95 (90–100)	0.308
PON1 general expression	Present	12 (92.4)	25 (96.2)	0.999
	Present focal	1 (7.6)	1 (3.89	
PON1 nuclear expression	Present	11 (84.6)	26 (100.0)	0.105
	Absent	2 (15.4)	0 (0.0)	

Abbreviations: HGUC, high-grade urothelial carcinoma; LGUC, low-grade urothelial carcinoma; MUSC, muscle-invasive urothelial carcinoma; PON1, paraoxonase-1; PPC, PON1-positive cells; TCSE, T1 subepithelial connective tissue invasion; TCSE NF, T1 subepithelial connective tissue invasion (non-flat).

**Table 2:** Biochemical and genetic analyses.

Parameter	Control Group (*n* = 47)	HGUC (*n* = 13)	*p*-Value ^1^	LGUC (*n* = 26)	*p*-Value ^2^	*p*-Value ^3^
Age	68 (60–79)	71 (56–85)	0.081	72 (45–87)	0.413	0.598
Male sex	47 (100.0)	12 (92.3)	0.217	25 (96.2)	0.356	1.000
PON1c, mg/L	99.9 (43.8–315.7)	75.7 (18.9–196.8)	0.134	91.6 (24.6–435.2)	0.475	0.677
Paraoxonase, U/L	278.9 (173.2–512.5)	224.0 (124.9–519.5)	0.125	245.9 (121.7–465.9)	0.413	0.251
Lactonase, U/L	5.8 (3.3–9.9)	3.8 (2.1–5.6)	<0.001	3.6 (1.8–6.1)	<0.001	0.777
PON1 192						
QQ	23 (48.9)	7 (53.8)		16 (61.6)		
QR	20 (42.6)	5 (38.5)	0.952	5 (19.2)	0.095	0.352
RR	4 (8.5)	1 (7.6)		5 (19.2)		
PON1 55						
LL	17 (36.2)	3 (23.1)		9 (34.6)		
LM	22 (46.8)	9 (69.2)	0.349	12 (46.1)	0.971	0.371
MM	1 (17.0)	1 (7.6)		5 (19.2)		

Abbreviations: HGUC, high-grade urothelial carcinoma; LGUC, low-grade urothelial carcinoma; PON1, paraoxonase-1. ^1^ HGUC vs. the control group; ^2^ LGUC vs. the control group; ^3^ LGUC vs. HGUC.

## Data Availability

The original contributions presented in this study are included in the article. Further inquiries can be directed to the corresponding author.

## Abbreviations

The following abbreviations are used in this manuscript:

HDL	High-density lipoproteins
HGUC	High-grade urothelial carcinoma
IHC	Immunohistochemistry
LACT	Lactonase activity
LGUC	Low-grade urothelial carcinoma
MTAP	Methylthioadenosine phosphorylase
MUSC	Muscle-invasive urothelial carcinoma
PARX	Paraoxonase activity
PON1	Paraoxonase-1
TBBL	5-thiobutyl butyrolactone
TCSE	T1 subepithelial connective tissue invasion
TCSE NF	T1 subepithelial connective tissue invasion (non-flat)
